# Gamma Irradiation Influences the Survival and Regrowth of Antibiotic-Resistant Bacteria and Antibiotic-Resistance Genes on Romaine Lettuce

**DOI:** 10.3389/fmicb.2019.00710

**Published:** 2019-04-09

**Authors:** Vaishali Dharmarha, Giselle Guron, Renee R. Boyer, Brendan A. Niemira, Amy Pruden, Laura K. Strawn, Monica A. Ponder

**Affiliations:** ^1^Department of Food Science and Technology, Virginia Tech, Blacksburg, VA, United States; ^2^Department of Civil and Environmental Engineering, Virginia Tech, Blacksburg, VA, United States; ^3^Food Safety and Intervention Technologies Research Unit, USDA-ARS Eastern Regional Research Center, Wyndmoor, PA, United States

**Keywords:** antibioitc resistance, *Eschericha coli* O157:H7, lettuce (*Lactuca sativa* L.), phyllo epiphytic microbiota, gamma irradiation, spoilage bacteria

## Abstract

Contamination of romaine lettuce with human pathogens, antibiotic-resistant bacteria (ARB), and antibiotic resistance genes (ARGs) occurs during production. Post-harvest interventions are emplaced to mitigate pathogens, but could also mitigate ARB and ARGs on vegetables. The objective of this research was to determine changes to lettuce phyllosphere microbiota, inoculated ARB, and the resistome (profile of ARGs) following washing with a sanitizer, gamma irradiation, and cold storage. To simulate potential sources of pre-harvest contamination, romaine lettuce leaves were inoculated with compost slurry containing antibiotic-resistant strains of pathogenic (*Escherichia coli* O157:H7) and representative of spoilage bacteria (*Pseudomonas aeruginosa*). Various combinations of washing with sodium hypochlorite (50 ppm free chlorine), packaging under modified atmosphere (98% nitrogen), irradiating (1.0 kGy) and storing at 4°C for 1 day versus 14 days were compared. Effects of post-harvest treatments on the resistome were profiled by shotgun metagenomic sequencing. Bacterial 16S rRNA gene amplicon sequencing was performed to determine changes to the phyllosphere microbiota. Survival and regrowth of inoculated ARB were evaluated by enumeration on selective media. Washing lettuce in water containing sanitizer was associated with reduced abundance of ARG classes that confer resistance to glycopeptides, β-lactams, phenicols, and sulfonamides (Wilcoxon, *p* < 0.05). Washing followed by irradiation resulted in a different resistome chiefly due to reductions in multidrug, triclosan, polymyxin, β-lactam, and quinolone ARG classes (Wilcoxon, *p* < 0.05). Irradiation followed by storage at 4°C for 14 days led to distinct changes to the β-diversity of the host bacteria of ARGs compared to 1 day after treatment (ANOSIM, *R* = 0.331; *p* = 0.003). Storage of washed and irradiated lettuce at 4°C for 14 days increased the relative abundance of *Pseudomonadaceae* and *Carnobacteriaceae* (Wilcoxon, *p* < 0.05), two groups whose presence correlated with detection of 10 ARG classes on the lettuce phyllosphere (*p* < 0.05). Irradiation resulted in a significant reduction (∼3.5 log CFU/g) of inoculated strains of *E. coli* O157:H7 and *P. aeruginosa* (ANOVA, *p* < 0.05). Results indicate that washing, irradiation and storage of modified atmosphere packaged lettuce at 4°C are effective strategies to reduce antibiotic-resistant *E. coli* O157:H7 and *P. aeruginosa* and relative abundance of various ARG classes.

## Introduction

The surface of green leafy vegetables, also known as the phyllopshere, is host to large populations of bacteria, with an of average 10^4^–10^8^ bacterial cells/cm^2^ (Morris and Kinkel, 2002; Leff and Fierer, 2013). While many of these bacteria are commensals contributing in plant growth or disease resistance, some may be plant pathogens or human pathogens (Lindow and Brandl, 2003; Dees et al., 2015). Between 1998 and 2018, 44 disease outbreaks were attributed to contamination of lettuce or foods containing lettuce with pathogenic strains of *Escherichia coli*, resulting in 1,358 illnesses, 394 hospitalizations, and 7 deaths in the United States (Centers for Disease Control and Prevention [CDC], 2018). Shiga toxin producing *Escherichia coli* (STEC) was the most common pathogen causing these outbreaks. Some outbreaks of *E. coli* illnesses and Shigellosis have been caused by strains that were resistant to multiple antibiotics, complicating treatment (Kapperud et al., 1995; Muniesa et al., 2012). For example, an outbreak of enterohemorrhagic *Escherichia coli* illnesses was traced back to fenugreek seed sprouts contaminated with multidrug resistant *E. coli* O104:H4, resulting in 2,987 illnesses, 855 cases of hemolytic uremic syndrome, and 53 deaths (Muniesa et al., 2012).

Contamination of leafy greens and other fresh produce may occur across the farm-to-fork continuum. Pre-harvest contamination through contact with biological soil amendments of animal origin (BSAAO) is a well-recognized source of human pathogens (Oliveira et al., 2012), and may also serve as a path for cross contamination with antibiotic-resistant bacteria (ARB) and antibiotic resistance genes (ARGs) in the farm environment (Marti et al., 2013). The abundance and mobility of ARGs is enriched in agricultural soils amended with manures and is correlated with transfer of ARGs to soil bacteria (Heuer and Smalla, 2007; Udikovic-Kolic et al., 2014). ARGs have been detected on harvested lettuce and endive after growing in manure-amended soils indicating that ARBs and ARGs on vegetables eaten raw may represent a direct route of dissemination of antibiotic resistance to bacteria in the human gut (Wang F.-H. et al., 2015; Wang L. et al., 2015). In the United States, the Food Safety Modernization Act (FSMA) Produce Safety Rule has set standards for BSAAO (US Food and Drug Administration [US FDA], 2015). It is recommended that animal manure be composted by acceptable treatment processes, as defined in §112.54, to achieve microbial standards, mainly pathogen reduction, as defined in §112.55 before it can be applied to land used for vegetable production (US Food and Drug Administration [US FDA], 2015). However, the effect of composting on ARGs is unclear. Composting may lead to reduction, enrichment, or have mixed effects on ARGs (Wang et al., 2012; Su et al., 2015; Qian et al., 2016; Tien et al., 2017). Application of compost to the soil may serve as a route of transfer of ARB and ARGs to food crops, but it may also reduce ARGs, emphasizing the need to also consider post-harvest interventions.

Lettuce is grown in close proximity to the soil, increasing the likelihood of becoming contaminated with a variety of ARB and ARGs (Holvoet et al., 2013; Marti et al., 2013; Wang F.-H. et al., 2015). Since high temperatures may damage sensory qualities of lettuce, thermal processing to reduce bacterial populations is not appropriate. However, several non-thermal interventions exist that have potential to reduce carriage of ARB and ARGs on vegetables surfaces interest. Common post-harvest practices include washing in water containing sanitizers, modified atmosphere packaging (MAP), and refrigerated storage are used to reduce bacterial numbers on produce surfaces, and increase the product shelf life in part due to reduced growth rates of spoilage (Beuchat et al., 1998; Parish et al., 2003; Keskinen et al., 2009). However, the ability of pathogens, such as *E. coli* O157:H7, to internalize into cut lettuce tissues, consequently reducing the efficacy of sanitizer washing (Takeuchi and Frank, 2000; Solomon et al., 2002), has warranted the approval of ionizing radiation (irradiation) for treatment of fresh lettuce and spinach for control of pathogens and shelf-life extension (US Food and Drug Administration [US FDA], 2008). Irradiation can be applied in the form of gamma rays and may have direct or indirect effects on bacterial DNA (Monk et al., 1995; Mañas and Pagán, 2005). While direct action results in the radiation energy deposition in DNA cells, indirect action primarily involves interaction of ionizing radiation with water molecules (Mañas and Pagán, 2005). Interaction of gamma rays with water produces reactive free radicals that damage DNA. Double strand breakage which cannot be repaired by the cells will result in cell death (Hall and Giaccia, 2006). Irradiation, singly and in combination with other treatments, can be calibrated for antimicrobial efficacy on a wide range of pathogens and commodities with minimal sensory impact to suspending foods, including leafy greens (Sommers et al., 2002; Alvarez et al., 2006; Niemira and Cooke, 2010; Lacombe et al., 2017). Gamma irradiation (0.25–1.5 kGy) is reported to reduce *E. coli* O157:H7, rifampicin-resistant *E. coli* and various members of the *Enterobacteriaceae* in several types of lettuce, but other heterotrophic bacteria remain (Niemira, 2008; Gomes et al., 2009; Osaili et al., 2018). ARB associated with plant surfaces may belong to a variety of phylogenetic groups (Holzel et al., 2018), including Gram positive bacteria (Micallef et al., 2013) whose susceptibility to gamma irradiation may be reduced compared to Gram negative bacteria (Halls, 1992). The diversity of these irradiation resistant bacteria in refrigerated MAP packaged lettuce is not well characterized. There is limited information on the effect of post-harvest technologies, including sanitizer washing, MAP, irradiation and refrigerated storage on the bacterial community dynamics of lettuce-associated ARB and ARGs.

The aim of this study was to determine the effects of post-harvest washing in sodium hypochlorite solution, irradiation, and cold storage (4°C) on the associated bacterial community of leaf lettuce, with a focus on the potential to mitigate ARB and ARGs that may be transferred to the plant from soil or biological amendments. Overall, this study provides insight into the possibility of various combinations of post-harvest practices, such as sanitizer washing, irradiation, MAP, and storage conditions as hurdles to the spread of antibiotic resistance via the food chain.

## Materials and Methods

### Experimental Design and Overview

To simulate potential sources of contamination, lettuce leaves were inoculated with a compost slurry containing ARB and ARGs originating from a biological amendment, in addition we included antibiotic-resistant strains of pathogenic *E. coli* O157:H7 and *Pseudomonas aeruginosa* as a representative of an important genus of concern for spoilage, as a known source of ARGs whose presence could be detected and quantified. The effects of post-harvest interventions on the corresponding lettuce leaf resistome (i.e., total ARG profile) was determined via shot-gun metagenomic sequencing. Corresponding changes to the relative abundance of taxonomic groups were assessed by 16S rRNA gene amplicon sequencing. Culture-based methods were used to quantify inoculated antibiotic-resistant *E. coli* O157:H7 and *P. aeruginosa*, with *tet*(A) carried by the latter strain further quantified by quantitative polymerase chain reaction (qPCR). Inoculated lettuce leaves were washed, packaged under MAP (98% nitrogen), subjected to gamma irradiation, and stored at 4°C for 14 days. Controls included: washed, non-irradiated lettuce (irradiation control), unwashed irradiated lettuce (wash control), washed, irradiated leaves inoculated with DNA of lysed *P. aeruginosa* containing 10^5^ copies of *tet*(A) gene (ARG DNA control). Experimental conditions included: sodium hypochlorite washed and non-irradiated lettuce stored for 1 day, sodium hypochlorite washed and non-irradiated lettuce and stored for 14 days, sodium hypochlorite washed and irradiated lettuce stored for 1 day, and sodium hypochlorite washed and irradiated lettuce stored for 14 days. Following these treatments, all lettuce was subject to MAP and stored at 4°C for 1 or 14 days.

### Bacterial Strains and Growth Conditions

Multi-drug resistant (MDR) *P. aeruginosa* strain PAO1 (ATCC 47085) and MDR *E. coli* O157:H7 strain SMS-3-5 (ATCC BAA-1743), both isolates from humans or the environment, and not produce associated, were revived from freezer stocks stored at -80°C by streaking onto Tryptic Soy Agar (TSA, Becton Dickinson, Franklin Lakes, NJ, United States) and incubating for 24 h at 37°C to obtain isolated colonies. An isolated colony of *P. aeruginosa* was streaked onto *Pseudomonas* Isolation Agar (PIA, Becton Dickinson, Franklin Lakes, NJ, United States) and an isolated colony of *E. coli* O157:H7 was streaked on to Eosin Methylene Blue Agar (EMB, Becton Dickinson, Franklin Lakes, NJ, United States) followed by incubation for 24 h at 37°C. Separate single colonies from PIA and EMB were incubated separately in Tryptic Soy Broth (TSB, Becton Dickinson, Franklin Lakes, NJ, United States) at 180 rpm for 24 h at 37°C. Cells were washed two times in 0.1% (wt/vol) peptone (Becton Dickinson, Franklin Lakes, NJ, United States) and were separately suspended in 9 ml of 0.1% (wt/vol) peptone to prepare the inoculation solution.

### Introduction of Compost-Associated Microorganisms to Lettuce

Compost generated from the manure of antibiotic-dosed cows, in a companion experiment (Ray et al., 2017), and was used to introduce compost-associated bacteria, including ARB and ARGs to lettuce. In brief, manure from dairy cattle was collected from cows that did not receive antibiotics (control dairy manure) or dairy cattle prophylactically administered cephapirin and therapeutic levels of pirlimycin (dairy manure with antibiotics). Both control dairy manure and dairy manure with antibiotics were collected and composted at 55°C for 3 days followed by storage at -20°C. Compost slurry was prepared by blending 50 g of DC or DCAB with sterile deionized water (450 ml for non-inoculated treatments or 440 ml for inoculated treatments) for 45 s using a blender (Oster^®^, Boca Raton, FL, United States). Experiments were conducted with three replicates of each type of slurry inoculum (DC or DCAB (*n* = 3), but because there were no statistically significant differences in the parameters examined in this study (total aerobic heterotrophic bacteria, relative abundance of bacterial communities and relative abundance of ARGs), all compost treatments were combined (referred as DC, dairy compost) for subsequent analysis (*n* = 6).

Romaine lettuce heads were obtained from a retail grocery store (Blacksburg, VA, United States). Damaged and cut leaves were removed. Remaining intact lettuce leaves were separated from the basal part, divided into 100 g portions and dip inoculated using two treatments: (1) Slurry generated from composted dairy manure (DC, dairy compost) (*n* = 6), and (2) The same DC slurry further spiked with the above described cocktail of MDR *P. aeruginosa* and *E. coli* (*n* = 3). To assure that quantifiable populations of known pathogens and spoilage-associated genera were introduced to the compost slurry, 10 ml of culture cocktail (containing 5 ml of *E. coli* O157:H7 and 5 ml of *P. aeruginosa*) was used to inoculate the compost slurry as described above. Inoculated leaves were air dried in a biological safety cabinet for approximately 15 min until visibly dry.

### Lettuce Washing and Modified Atmosphere Packaging

Inoculated and dried lettuce leaves were washed with sodium hypochlorite (XY-12, EcoLab) per manufacturer’s recommendation (50 ppm free chlorine) with a 2 min contact time followed by a 30 s rinse with tap water. Free chlorine was measured for XY-12 washes and tap water rinse using free chlorine test strips (Chlorine Test Paper, Ecolab). Excess liquid was removed by drying using a salad spinner followed by air drying for 15 min in a biological safety cabinet. Whole leaves were then packaged under 98% nitrogen (Sandhya, 2010) in PD961 EZ film bags (Cryovac, Duncan, SC; oxygen transmission rate: 6000–8000 mL/m^2^/24 h) using the Koch Ultravac vacuum packaging machine. A set of unwashed lettuce was retained, which served as a wash control.

### Irradiation, Refrigerated Storage, and Headspace Gas Analysis

MAP lettuce was maintained at 4^o^C and shipped to the USDA Food Safety and Interventions Technologies Research Unit (Wyndmoor, PA, United States) where it was treated with 1.0 kGy gamma irradiation at 4°C using a Cs-137 self-contained gamma radiation source (Lockheed-Georgia, Marietta, GA, United States) at 3.95 kGy/h. Irradiated lettuce was returned to Virginia Tech where it was stored at 4^o^C for 14 days. Controls of non-irradiated washed and non-irradiated unwashed lettuce were also stored under the same conditions. Lettuce samples were prepared for analysis on day 1 (3 days after inoculation, including transit time) and on day 14 (17 days after inoculation, including transit time). Before opening the packages for microbiological analysis on days 1 and 14, oxygen and carbon dioxide levels of the packages were determined using Mocon Dansensor CheckPoint Handheld Gas Analyzer (Mocon Inc., Minneapolis, MN, United States).

### Culture Dependent Analysis

#### Media Preparation

EMB, PIA, and R2A were prepared according to manufacturer’s directions (BD, Franklin Lakes, NJ, United States). Stock solution of tetracycline (Dot Scientific, Burton, MI, United States) was added to PIA to achieve a final concentration of 4 μg/ml of tetracycline in PIA. This tetracycline concentration corresponds to the MIC of the inoculated *Pseudomonas aeurginosa* and therefore was chosen to select for *Pseudomonas* sp. resistant to tetracycline.

#### Microbiological Analysis

Total aerobic heterotrophic bacteria, inoculated *E. coli* O157:H7 and *Pseudomonas* sp. were enumerated by plate counts. Treated or control lettuce leaves (100 g) were removed from MAP, transferred into a sterile filter bag (Fisher Scientific), and suspended in sterile peptone tween 80 buffer (PT, both 0.1% w/v) in order to achieve a 1:10 dilution. The bag was then shaken at a speed of 220 rpm for 5 min using a bench top rotator, subsequently hand-massaged for 2 min, and then shaken again using the bench top rotator for an additional 5 min. The liquid from sterile filter bags was mixed. A 1 ml aliquot was drawn and serially diluted in 0.1% peptone after which 100 μl was spread on duplicate plates of each media type and incubated for 18–24 h at 37°C.

### Culture Independent Analyses

#### DNA Extraction

The remaining liquid contents (∼1 L) of each bag were centrifuged at 1,096 × *g* for 20 min to reduce the amount of visible chloroplast. The supernatant was separated without disturbing the pellet and vacuum filtered through a 0.22 μm, 47 mm mixed cellulose ester membrane (Millipore Sigma, Burlington, MA, United States) filter to collect bacterial cells from the lettuce surface. Filters were stored within sterile, DNase-free, O-ring screwcap tubes at -80°C until DNA extractions were performed. DNA extractions were performed using FastDNA^®^ Spin Kit for Soil (MP Biomedical^TM^, Solon, OH, United States) per manufacturer’s instructions with an additional bead beating step and incubation for 2 h to facilitate lysis of bacterial cells. The DNA was eluted in 100 μl of DNase/pyrogen free water and was subsequently treated with a OneStep^TM^ PCR Inhibitor Removal Kit (Zymo Research, Irvine, CA, United States) per manufacturer’s instructions. DNA was stored at -80°C until qPCR was performed.

#### Quantification of 16S rRNA Gene and *tet*(A) Gene

Quantification of 16S rRNA genes (Suzuki et al., 2000) and the *tet*(A) gene (Ng et al., 2001) copy numbers from lettuce DNA samples was performed using qPCR. DNA extracts from unwashed samples were diluted 1:10 in order to reduce PCR inhibition. Each reaction mixture (10 μl) consisted of SsoAdvanced^TM^ Universal SYBR Green Supermix (Bio-Rad Laboratories, Hercules, CA, United States), 400 nM forward primer, 400 nM reverse primer, 20–76 ng DNA template, and molecular grade water (Sigma-Aldrich). The protocol on CFX Connect^TM^ (Bio-Rad Laboratories, Hercules, CA, United States) consisted of 1 cycle of 98°C for 2 min, 40 cycles of 98°C for 5 s and annealing temperature at 55^o^C for 5 s (16S rRNA gene) and 66^o^C for 30 s [*tet*(A) gene] followed by a melt curve. Standard curves were created using seven 10-fold dilutions of 16S rRNA gene and *tet*(A) gene (10^8^–10^2^ copies/μl).

#### 16S rRNA Gene Amplicon and Shotgun Metagenomic Sequencing

DNA samples from each lettuce treatment were diluted to 3 × 10^6^ 16S rRNA gene copies/μl to account for variability in 16S rRNA gene copies or extraction efficiency differences among the treatments. The 16S rRNA gene amplicon sequencing for bacterial communities was based on the Earth Microbiome Project protocol using primers 515fB and 926r (Walters et al., 2016). Each DNA sample was amplified using individually barcoded forward primer. Each reaction mixture (25 μl) consisted of 2.5× 5PRIME HotMaster Mix (QuantaBio, Beverly, MA, United States), 515fB primer (10 μM), 926r primer (10 μM), molecular grade water and template DNA. The protocol was run on the CFX Connect^TM^ (Bio-Rad Laboratories, Hercules, CA, United States) and thermocycler conditions were 1 cycle of 94°C for 3 min, 35 cycles of 94°C for 45 s, 50°C for 60 s, and 72°C for 90 s, and a final extension cycle at 72°C for 10 min. Amplicons for each lettuce treatment were quantified after PCR amplification using a Qubit 3.0 Fluorometer (Thermo Fisher Scientific, Waltham, MA, United States). Amplicons (240 ng) with unique barcodes were then pooled together in a single tube for 16S rRNA amplicon sequencing. Libraries were prepared at Virginia Tech Biocomplexity Institute (Blacksburg, VA, United States) using the MiSeq^®^ Reagent Kit v3 (Illumina) and 2 × 300 bp paired-end reads were produced using MiSeq (Illumina). Raw reads were deposited into the Sequence Read Archive (SRP 158273). Paired end reads were stitched using PANDASeq (Masella et al., 2012) and filtered based on the quality score (≥0.90) and sequence length (372–375 bp). Chimera sequences were removed using Chimera slayer with QIIME (Haas et al., 2011). Chloroplast and mitochondrial sequences were removed using QIIME. Operational taxonomic units (OTUs) were assigned *de novo* using QIIME from the GreenGenes database at 97% cutoff (Caporaso et al., 2011). OTU libraries derived from each lettuce sample were rarefied to 1,000 reads using QIIME (single_rarefaction.py), equivalent to the sample with the shallowest sequencing depth, before performing diversity analyses. Shannon index and Chao1 were selected for the alpha diversity matrix, while Unifrac distance was selected for β-diversity (Shannon and Weaver, 1949; Chao, 1984; Lozupone and Knight, 2005).

To determine the effect of irradiation on relative abundance of ARGs after a period of time reflective of a typical lettuce shelf life, shotgun metagenomic sequencing was performed for day 14 irradiated and non-irradiated lettuce samples. Samples were prepared using the NEBNext Ultra II DNA library prep (New England Biolabs, Ipswich, MA, United States) using 9-PCR cycles and sequenced on an Illumina NextSeq 500 75 bp paired-end protocol at Scripps Research Institute (San Diego, CA, United States). Merging of paired-end reads and quality control was performed using TRIMMOMATIC to remove low quality sequences from the dataset (Arango-Argoty et al., 2016). Bowtie2 (Langmead and Salzberg, 2012) was used to remove chloroplast sequences from the metagenomics sequence reads. Chloroplast sequences were downloaded from Greengenes (May 2013 release). To account for variability in sequencing depth among the samples, the number of sequences obtained from each sample after chloroplast filtering was subsampled to 14,998,741 reads using Seqtk. Scripts can be found at https://github.com/gaarangoa/genomic-scripts. Resulting sequence files were uploaded onto MetaStorm^1^ (Arango-Argoty et al., 2016), a webserver configured for environmentally-derived metagenomic sequencing data, which is accessible to the public. From rarefied shotgun metagenomics reads, ARGs were aligned to the Comprehensive Antibiotic Resistance Database (version 1.2.1) (*e*-value < 1e-10, identity > 80%, and ≥ 25 amino acids) (Jia et al., 2017). For constructing a heat map to identify correlations between bacterial families and ARGs on lettuce, bacterial families from metagenomics reads were annotated using Greengenes (DeSantis et al., 2006) database with a 90% cut off and minimum alignment length of 25aa. Relative abundance of ARGS was determined by normalizing the number of matches using the 16S rRNA normalization of each gene.

#### Statistical Analysis

Statistical analyses were performed using JMP statistical software (version 13, SAS, Cary, NC, United States). The bacterial CFU/g were log transformed to achieve normal distribution. The effects of irradiation (irradiated or non-irradiated) by days of storage (Days 1 and 14) were compared using ANOVA with a Fisher’s *post hoc* analysis to test for differences in the mean log CFU/g of aerobic heterotrophic bacteria, *E. coli* O157:H7 and *Pseudomonas* resistant to 4 μg/ml tetracycline. *P* < 0.05 was applied to designate significance.

Comparison of the relative abundances of bacterial taxonomic groups from the 16S rRNA gene sequencing was performed using non-parametric Wilcoxon tests due to non-normal distribution. No significant differences between relative abundance of taxonomic groups on inoculated and non-inoculated lettuce were found, therefore replicates were pooled for statistical power for subsequent analysis of lettuce phyllosphere bacterial communities (*n* = 9). Relative abundance of taxonomic groups on irradiated washed samples were compared with non-irradiated washed samples on day 14 to determine the effect of irradiation. Relative abundance of taxonomic groups on unwashed irradiated samples were also compared with washed irradiated samples on day 14 to determine the effect of washing prior to irradiation. Relative abundances of taxa at phylum, class, order, family, and genus level were determined. *P* < 0.05 were used to designate significance. Analysis of β-diversity was via non-metric multidimensional scaling (NMDS) plots using weighted Unifrac distances by each treatment type and determination of analysis of similarity (ANOSIM) using Primer (version 6.1.13, Plymouth, United Kingdom). *R*-value cutoffs as defined by Clarke and Warwick (2006) were used (*R* > 0.75, well separated; *R* > 0.5, separated but overlapping; *R* < 0.25, barely separated) and *p* < 0.05 were used to assess separation between bacterial communities across treatments.

Statistical analysis of relative abundances of ARGs on Day 14 samples was performed using non-parametric Wilcoxon tests. Since no significant differences in relative abundance of ARGs on inoculated and non-inoculated lettuce were detected, replicates were pooled for subsequent analysis (*n* = 9). Relative abundance of ARGs on irradiated washed samples were compared with non-irradiated washed samples on day 14 to determine the effect of irradiation. Relative abundance of ARGs on unwashed irradiated samples were also compared with washed irradiated samples to determine the effect of washing. *P* < 0.05 were used to designate significance. Primer (version 6.1.13, Plymouth, United Kingdom) was used to determine ANOSIM and to generate NMDS plots based on Bray-Curtis distances obtained from metagenomics ARG relative abundances. The relative dissimilarity of ARGs in samples from different treatments was defined as separable from others when *R* > 0.25 and *p* < 0.05. To analyze the correlations between ARGs and bacterial families on lettuce, Spearman’s rank correlation coefficients were calculated in JMP statistical software (version 13, SAS, Cary, NC, United States). *P* < 0.05 was used to designate significance.

## Results

### Bacterial Communities on Lettuce Phyllosphere

16S rRNA gene amplicon sequences obtained from unwashed lettuce were assigned to 16 phyla. Over 92% of sequences were classified (in order of most abundant) within only three phyla: *Proteobactetria, Firmicutes, and Actinobacteria* (Supplementary Table S1). Within the classified phyla, 29 bacterial classes were identified and ∼97% of sequences (in order of most abundant) belonged to the classes *Bacilli, Gammaproteobacteria, Actinobacteria, Clostridia, Betaproteobacteria*, and *Alphaproteobacteria* (Supplementary Table S1). From the classified sequences, 46 bacterial orders were identified. Bacterial orders with about 77% of sequences (from most to least abundant) belonged to *Bacillales*, *Pseudomonadales*, *Actinomycetales, Methylophilales, Enterobacteriales*, and *Clostridiales* (Supplementary Table S1). Overall 90 bacterial families were identified and 53% sequences were classified in *Pseudomonadaceae*, *Planococcaceae, Nocardiospaceae, Methylophilaceae*, *Enterobacteriaceae*, and *Bacillaceae.* Most represented bacterial genera (in order of abundance) belonged to *Pseudomonas, Ureibacillus, Escherichia, Bacillus, Symbiobacterium*, and *Thermoactinomycetes*. Together they constituted about 37% of the classified sequences in the genera category (Supplementary Table S1).

### Irradiation Altered Phyllosphere Bacterial Community Structure on Romaine Lettuce

**FIGURE 1:**
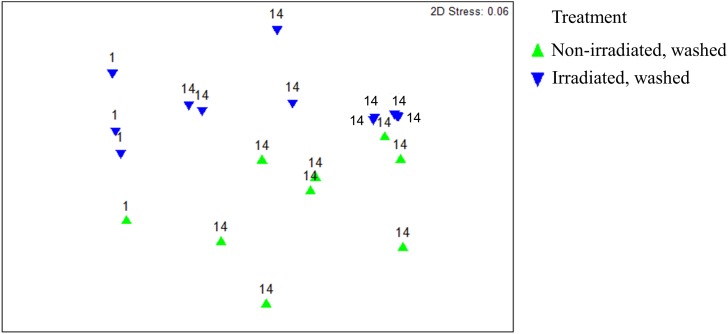
Non-metric multidimensional scaling analysis of weighted Unifrac distance matrices of surficial bacterial microbiota found on irradiated and non-irradiated lettuce surfaces after 1 and 14 days of storage based on 16S rRNA gene amplicon sequencing. Each point represents one lettuce sample. ANOSIM, *R* = 0.171, *p* = 0.036 indicates irradiated and non-irradiated lettuce had overall similar bacterial community structures (ANOSIM, *R* > 0.25 and *p* < 0.05). ANOSIM, *R* = 0.673, *p* = 0.002 indicates distinct bacterial community structures on lettuce surfaces stored for 1 vs. 14d.

Overall, β-diversity of irradiated and non-irradiated lettuce was similar, immediately after irradiation (ANOSIM, *R* = 0.171, *p* = 0.002; Figure 1). However, after 14 days storage at 4°C the bacterial community profiles of irradiated and non-irradiated samples were significantly distinct, ANOSIM, *R* = 0.673, *p* = 0.002, Figure 1), indicating that re-growth of bacteria surviving the 1.0 kGy dose alters the community structure during subsequent storage. Bacterial community richness and evenness (Chao1, Shannon) were unchanged immediately after treatment, but significantly declined during the 14 days refrigerated storage period (Supplementary Table S2). Analysis of relative abundance of bacterial taxa indicated significant shifts in bacterial community composition of irradiated lettuce after 14 days of storage compared to non-irradiated lettuce (Table 1). There were no statistical differences in relative abundance of bacterial taxa between non-irradiated and irradiated day 1 lettuce samples, therefore the averaged day 1 samples were compared to day 14 non-irradiated and irradiated lettuce samples (Table 1). Irradiation resulted in a 30% decrease in *Proteobacteria* and a 26% increase in *Firmicutes* compared to non-irradiated lettuce stored for 14 days (*p* < 0.020, Wilcoxon, Table 1). Relative abundance of *Gammaproteobacteria*, which includes the inoculated antibiotic resistant pathogen and spoilage bacteria in this study, decreased significantly (30%) on irradiated lettuce compared to the non-irradiated lettuce after 14 days of storage (*p* < 0.001, Wilcoxon). A comparison of the relative abundance of the lettuce phyllosphere communities for Day 1 and irradiated Day 14 lettuce indicated a 40.2% increase in *Bacilli* and 17% decrease in *Gammaproteobacteria* after 14 days of storage (*p* < 0.026, Wilcoxon). No significant changes in *Bacilli* and *Gammaproteobacteria* were observed between Day 1 and non-irradiated Day 14 lettuce samples, indicating that irradiation was responsible for altering the bacterial communities that regrew during the 14 days period (Table 1). Storage for 14 days was associated with an increase in relative abundance of *Carnobacteriaceae* by 50.3% on non-irradiated lettuce and 62.2% on irradiated lettuce (*p* < 0.009, Wilcoxon). *Pseudomonadaceae* showed a 12.5% decline from days 1 to 14 irradiated samples (*p* = 0.020, Wilcoxon), while no significant differences were noted between day 1 and non-irradiated day 14 samples, indicating that irradiation was effective in preventing regrowth of part of the bacterial community (Table 1). Bacterial genera that decreased significantly (*p* < 0.050, Wilcoxon) in relative abundance on irradiated lettuce compared to non-irradiated lettuce on 14 days included: *Pseudomonas* (reduced by 20%), *Yersinia* (reduced by 6%), and genera of unclassified *Enterobacteriaceae* (reduced by 4%). In comparing samples immediately after irradiation, it is evident that the relative abundance of *Pseudomonas* decreased further during storage for 14 days (*p* = 0.021, Wilcoxon), indicating that the combination of post-harvest interventions was effective in reducing these potential spoilage bacteria. Y*ersinia* was not detected on 1 day lettuce, yet its relative abundances climbed to 6.3% on non-irradiated lettuce at 14 days. Relative abundance of unclassified *Enterobacteriaceae* increased from 0.25% on 1 day samples to 4.3% in non-irradiated lettuce stored for 14 days. In contrast, the relative abundance of *Yersinia* and genera of unclassified *Enterobacteriaceae* did not increase in the irradiated lettuce stored for 14 days lettuce (Table 1).

**Table 1:** Relative abundance (%) of bacterial groups of the washed lettuce phyllosphere whose membership is significantly affected by irradiation and cold storage.

Treatment Taxa	Day 1^∗^	Non-irradiated Day 14	Irradiated Day 14
**PHYLUM**
*Proteobacteria*	25.95^A^	35.28^AB^	5.70^C^
*Firmicutes*	42.00^A^	60.69^AB^	86.74^C^
*Actinobacteria*	27.33^A^	2.73^C^	5.84^C^
*Other*	4.73^A^	1.30^C^	1.72^C^
**CLASS**
*Bacilli*	38.38^A^	59.54^AC^	78.57^C^
*Gammaproteobacteria*	19.63^A^	33.19^A^	2.61^C^
*Actinobacteria*	27.23^A^	2.73^C^	5.81^C^
*Clostridia*	3.70^A^	1.14^BC^	8.10^AC^
*Betaproteobacteria*	3.63^A^	1.43^BC^	1.52^AC^
*Alphaproteobacteria*	2.08^A^	0.43^BC^	1.17^AC^
*Other*	5.48^A^	1.54^BC^	2.22^AC^
**ORDER**
*Lactobacillales*	1.95^A^	53.41^BC^	63.39^C^
*Bacillales*	36.05^A^	5.73^BC^	12.49^C^
*Pseudomonadales*	14.08^AB^	21.30^B^	4.79^A^
*Actinomycetales*	27.23^A^	2.73^C^	5.71^C^
*Enterobacteriales*	1.00^A^	11.08^B^	3.29^A^
*Clostridiales*	3.30^A^	1.09^BC^	3.71^AC^
*Methylophilales*	1.28^A^	0.54^A^	0.86^A^
*Xanthomonadales*	2.85^A^	0.63^BC^	0.81^C^
*Burkholderiales*	2.30^A^	0.86^AC^	0.66^C^
*Alteromonadales*	1.13^A^	0.08^BC^	0.32^AC^
*Other*	8.85^A^	2.58^BC^	3.98^AC^
**FAMILY**
*Carnobacteriaceae*	1.93^A^	52.26^C^	64.13^C^
*Pseudomonadaceae*	13.38^A^	21.20^A^	0.87^C^
*Planococcaceae*	17.28^A^	2.51^C^	5.31^C^
*Nocardiopsaceae*	22.78^A^	1.76^BC^	4.54^AC^
*Enterobacteriaceae*	1.00^A^	11.08^BC^	0.32^AC^
*Bacillaceae*	5.85^A^	0.80^BC^	1.99^AC^
*Bacillales, other*	4.45^A^	0.75^BC^	1.93^AC^
*Methylophilaceae*	1.28^A^	0.54^A^	0.99^A^
*Paenibacillaceae*	3.50^A^	0.59^BC^	1.71^AC^
*Thermoactinomycetaceae*	3.15^A^	0.71^BC^	2.11^AC^
*Clostridiaceae*	0.30^A^	0.30^A^	5.74^A^
*Symbiobacteriaceae*	1.80^A^	0.46^BC^	1.64^AC^
*Xanthomonadaceae*	2.33^A^	0.58^BC^	0.76^C^
*Other*	21.00^A^	6.46^BC^	7.94^C^
**GENUS**
*Carnobacterium*	1.80^A^	52.19^BC^	64.09^C^
*Pseudomonas*	13.13^A^	21.13^A^	0.80^C^
*Ureibacillus*	13.58^A^	2.03^BC^	4.49^C^
*Bacillus*	5.30^A^	0.66^BC^	1.68^C^
*Clostridium*	0.25^A^	0.30^A^	5.48^A^
*Symbiobacterium*	1.80^A^	0.46^A^	1.64^A^
*Yersinia*	0.00^A^	6.35^B^	0.01^A^
*Thermoactinomyces*	2.05^A^	0.51^A^	1.46^A^
*Enterobacteriaceae, other*	0.25^A^	4.24^B^	0.03^A^
*Escherichia*	0.68^A^	0.08^A^	0.21^A^
*Other*	61.18^A^	12.06^BC^	20.11^C^


**^∗^No statistical differences in the relative abundance of bacterial taxa between non-irradiated and irradiated Day 1 lettuce samples were found, therefore average of Day 1 irradiated and non-irradiated lettuce samples were compared to Day 14 non-irradiated and irradiated lettuce samples. Connecting letters (A,B, and C) denote statistical comparison between relative abundance of bacterial members of lettuce phyllosphere on non-irradiated Day 1 versus non-irradiated Day 14 lettuce; irradiated Day 1 versus irradiated Day 14 lettuce; and non-irradiated day 14 versus irradiated day 14 lettuce (Wilcoxon, p < 0.05). Other corresponds to all bacterial groups at each taxonomic level with relative abundance of less than 1%.**

The effect of irradiation on the ARG *tet*(A) that was contained within an intact microorganism (inoculated *P. aeruginosa*) or in naked DNA of the non-intact microorganism, was quantified using qPCR. No detectable copies of *tet*(A) were detected on samples on non-inoculated lettuce. Relative abundance of *tet*(A) genes normalized for the 16S rRNA genes measured in washed non-irradiated lettuce stored for 1 day was 1.45 × 10^-4^*±* 1.35 × 10^-4^ *tet*(A) gene copies/16S rRNA gene copies and did not change significantly after irradiation or storage of irradiated lettuce for 14 days (Supplementary Table S3). The absolute abundance of *tet*(A) (total *tet*(A) gene copies) in washed non-irradiated lettuce stored for 1 day was 1.56 × 10^4^ ± 6.04 × 10^3^. Thus, irradiation did not result in measureable changes to absolute abundance of *tet*(A) within intact cells or naked DNA on lettuce stored for 1 or 14 days (Supplementary Table S4). Storage of irradiated and washed lettuce over 2 weeks at 4^o^C resulted in what appeared to be a small, but not statistically significant, reduction in absolute and relative abundance of *tet*(A) (Supplementary Tables S3, S4). There was no significant difference in relative abundance of *tet*(A) detected from treatments featuring intact cells versus naked DNA.

### Irradiation Reduced Survival and Re-growth of Antibiotic-Resistant Bacteria on Romaine Lettuce

**FIGURE 2:**
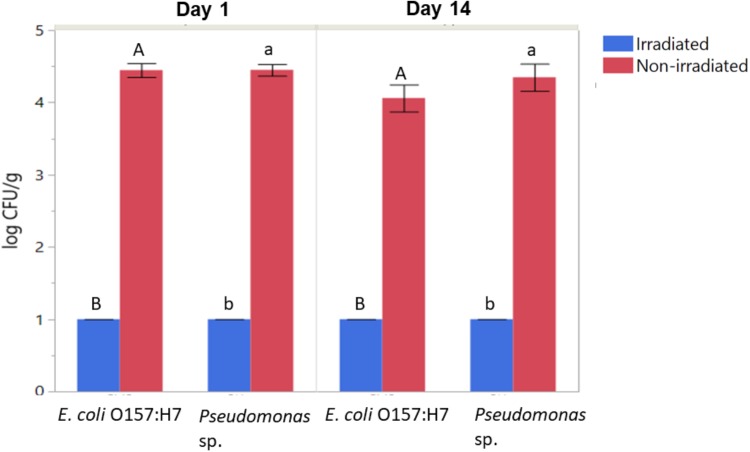
Effect of irradiation on the survival and regrowth of inoculated antibiotic resistant E. coli O157:H7 and Pseudomonas after 1 and 14 days of storage at 4°C. Bars represent average log CFU/g and error bars represent standard error from the mean (*n* = 3). Connecting letters indicate statistical significance among irradiated and non-irradiated samples as well as among days 1 and 14 samples (ANOVA, *p* < 0.05).

Populations of aerobic heterotrophic bacteria reduced from 4.70 ± 0.26 to 2.75 ± 0.88 log CFU/g following irradiation and 14 days of storage, indicating effectiveness of irradiation in reducing the total aerobic populations on romaine lettuce and that the populations did not return to initial levels during the 14 days of storage. In contrast, the total aerobic heterotrophic bacteria count for the non-irradiated control had bacterial counts 4.89 ± 0.08 log CFU/g. In particular, irradiation combined with washing and refrigerated storage resulted in significant reductions in the culturable log CFU/g of inoculated *E. coli* and *Pseudomonas* (Figure 2, ANOVA, *p* < 0.05). *E. coli* O157:H7 and *Pseudomonas* populations were decreased from ∼4.5 log CFU/g on non-irradiated lettuce to below the limit of detection (BLD; 2 log CFU/g) on irradiated lettuce (ANOVA, *p* < 0.05). No declines of inoculated *E. coli* O157:H7 or *Pseudomonas* sp. were observed on non-irradiated lettuce stored for 14 days (Figure 2). No significant regrowth of either inoculated bacteria occurred on irradiated lettuce during the 14 days of storage (Figure 2). This is consistent with 16S rRNA sequencing results, where no significant changes to the relative abundance of OTUs classified as *Escherichia* were observed between samples 1 day versus 14 days after irradiation. Due to the presence of substantial numbers of native *Pseudomonas* sp. on the lettuce phyllosphere, the use of antibiotic in culturing assays was necessary to facilitate detection of the inoculated strain. While irradiation did result in a loss in culturability of the inoculated *Pseudomonas* strain, other native members of the *Pseudomonas* population increased during the 14 days of storage post-irradiation (Table 1). While the antibiotic resistance statuses of the native strains cannot be confirmed, there were no colonies detected on PIA tetracycline plates from non-inoculated lettuce, indicating irradiation decreased the inoculated strain of *Pseudomonas*.

### Washing Lettuce Prior to Irradiation Resulted in Larger Shifts in Bacterial Community Composition During Storage

Inclusion of sanitizer lettuce wash water significantly decreased the bacterial community richness and evenness (Chao1, Shannon) compared to unwashed lettuce (Supplementary Table S2). Relative abundance of some bacterial taxa on the washed irradiated lettuce phyllosphere stored for 14 days were significantly different compared to the unwashed lettuce (Table 2). Most notably, there was a 36.4% decline in relative abundance of *Proteobacteria* and a 47% increase in relative abundance of *Firmicutes* when washing with sanitizer was employed prior to irradiation (Wilcoxon, *p* = 0.045, Table 2). *Bacilli*, a representative class of *Firmicutes*, increased by 46.0% and *Proteobacteria* classes- *Gammaproteobacteria, Betaproteobacteria*, and *Alphaproteobacteria*, were reduced by 27.9, 8.83, and 3.98%, respectively, after washing (Wilcoxon, *p* < 0.05, Table 2). Washing and storage for 14 days were associated with the increase in relative abundance of *Carnobacterium* by 63.6%, while the relative abundance of *Pseudomonas* and *Escherichia* decreased by 15.3 and 1.9%, respectively, compared to unwashed irradiated lettuce (Wilcoxon, *p* < 0.05, Table 2).

**Table 2:** Inclusion of sodium hypochlorite sanitizer in wash water prior to irradiation and cold storage for 14 days significantly affects relative abundance of some bacterial groups of the lettuce phyllosphere.

Treatment Taxa	Irradiated unwashed day 14	Irradiated washed day 14
**PHYLUM**
*Proteobacteria*	42.05^A^	5.70^B^
*Firmicutes*	39.25^A^	86.74^B^
*Actinobacteria*	9.20^A^	5.84^A^
*Other*	9.50^A^	1.71^A^
**CLASS**
*Bacilli*	32.55^A^	78.57^B^
*Gammaproteobacteria*	25.25^A^	2.61^B^
*Actinobacteria*	9.15^A^	5.81^A^
*Clostridia*	6.65^A^	8.10^A^
*Betaproteobacteria*	10.35^A^	1.52^B^
*Alphaproteobacteria*	5.15^A^	1.17^B^
*Other*	10.90^A^	2.22^B^
**ORDER**
*Lactobacillales*	0.45^A^	63.39^B^
*Bacillales*	32.00^A^	12.49^A^
*Pseudomonadales*	16.85^A^	4.79^A^
*Actinomycetales*	9.15^A^	5.71^A^
*Enterobacteriales*	2.15^A^	3.29^A^
*Clostridiales*	6.15^A^	3.71^A^
*Methylophilales*	6.80^A^	0.86^B^
*Xanthomonadales*	3.85^A^	0.81^A^
*Burkholderiales*	3.00^A^	0.66^A^
*Alteromonadales*	1.95^A^	0.32^B^
*Other*	17.65^A^	3.98^B^
**FAMILY**
*Carnobacteriaceae*	0.45^A^	64.13^B^
*Pseudomonadaceae*	16.45^A^	0.87^B^
*Planococcaceae*	12.20^A^	5.31^A^
*Nocardiopsaceae*	6.00^A^	4.54^A^
*Enterobacteriaceae*	2.15^A^	0.32^B^
*Bacillaceae*	5.50^A^	1.99^A^
*Bacillales, other*	5.30^A^	1.93^A^
*Methylophilaceae*	6.80^A^	0.99^B^
*Paenibacillaceae*	4.40^A^	1.71^A^
*Thermoactinomycetaceae*	3.50^A^	2.11^A^
*Clostridiaceae*	0.20^A^	5.74^A^
*Symbiobacteriaceae*	4.25^A^	1.64^A^
*Xanthomonadaceae*	3.25^A^	0.76^A^
*Other*	29.55^A^	7.94^B^
**GENUS**
*Carnobacterium*	0.45^A^	64.09^B^
*Pseudomonas*	16.05^A^	0.80^B^
*Ureibacillus*	9.75^A^	4.49^A^
*Bacillus*	4.55^A^	1.68^A^
*Clostridium*	0.15^A^	5.48^A^
*Symbiobacterium*	4.25^A^	1.64^A^
*Yersinia*	0.00^A^	0.01^A^
*Thermoactinomyces*	2.40^A^	1.46^A^
*Enterobacteriaceae, other*	0.00^A^	0.03^A^
*Escherichia*	2.10^A^	0.21^B^
*Other*	60.30^A^	20.11^B^


*Connecting letters (A and B) denote statistical comparison between relative abundance of bacterial members of lettuce phyllosphere on irradiated unwashed and washed Day 14 lettuce (Wilcoxon, p < 0.05). Other corresponds all bacterial groups at each taxonomic level with relative abundance of less than 1%.*

### Washing in Combination With MAP Reduced Survival and Regrowth of Antibiotic-Resistant Bacteria on Romaine Lettuce

**FIGURE 3:**
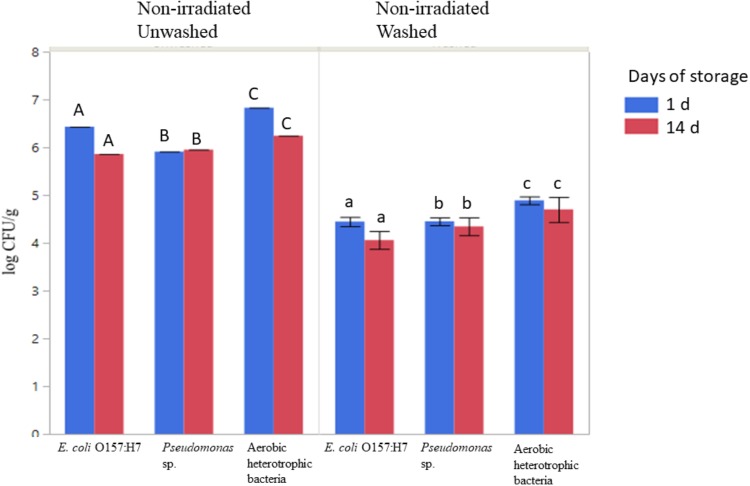
Effect of washing on the survival and regrowth of inoculated antibiotic resistant *E. coli* O157:H7, *Pseudomonas*, and aerobic heterotrophic bacteria after 1 and 14 days of storage at 4°C. Bars represent average log CFU/g and error bars represent standard error from the mean (*n* = 3). Connecting letters indicate statistical significance among non-irradiated unwashed and non-irradiated washed samples as well as among days 1 and 14 samples (ANOVA, *p* < 0.05).

The populations of aerobic heterotrophic bacteria decreased from 6.53 ± 0.39 to 4.60 ± 0.13 log CFU/g following washing with sanitizer (sodium hypochlorite, 50 ppm free chlorine), packaging under modified atmosphere, and storage at 4°C for 1 or 14 days (ANOVA, *P* < 0.05, Figure 3). No significant regrowth of aerobic heterotrophic bacteria was observed on sanitizer-washed lettuce in MAP after 14 days of storage at 4°C (Figure 3). Sanitizer washing combined with MAP and storage at 4°C resulted in significant reductions in the culturable log CFU/g of inoculated antibiotic-resistant pathogenic and spoilage bacteria (Figure 3; ANOVA, *P* < 0.05). The *E. coli* O157:H7 population decreased by ∼ 2 log CFU/g on sanitizer-washed lettuce compared to unwashed lettuce (Figure 3; ANOVA, *p* < 0.05). Sanitizer washing also reduced *Pseudomonas* sp. resistant to tetracycline on lettuce from ∼5.9 log CFU/g on unwashed lettuce to ∼4.4 log CFU/g on sanitizer-washed lettuce. No significant regrowth of either inoculated bacteria occurred on unwashed or sanitizer-washed lettuce during the 14 days of storage indicating MAP and storage at 4°C was effective in suppressing the bacterial growth on sanitizer-washed lettuce over time (Figure 3).

### Correlations Between Phyllosphere ARGs and Bacterial Community Members

**FIGURE 4:**
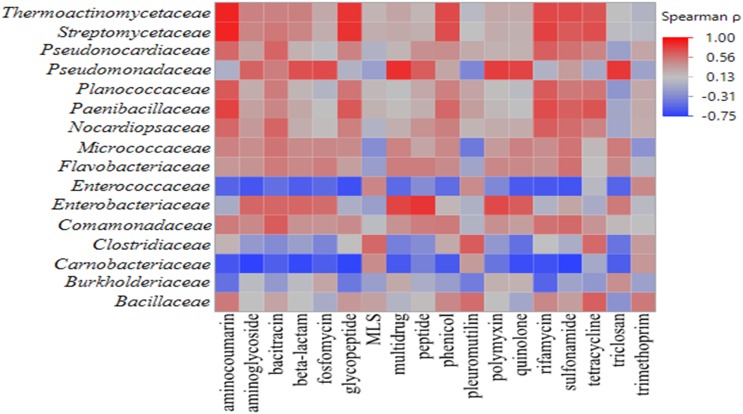
Heat map of Spearman’s rank correlation coefficients obtained when comparing relative abundances of bacterial families and ARG classes detected in the irradiated and non-irradiated lettuce phyllospheres via 16S rRNA gene amplicon sequencing and shotgun metagenomic sequencing, respectively (*n* = 22). Intensity of shading from blue to red indicates strength of correlation from negative to positive, respectively.

Across all samples, 81,845 reads aligned to 577 ARGs were identified on romaine lettuce. Based on the mechanisms of resistance, detected ARGs were categorized into 22 classes. Genes conferring resistance to antibiotic drug classes glycopeptide, macrolide-lincosamide-streptogramin, multidrug, polymyxin, rifamycin, tetracycline, triclosan, and trimethoprim were detected in all samples. Spearman’s rank correlation analysis revealed significant correlations among bacterial families and ARGs found on the lettuce phyllosphere (Figure 4 and Supplementary Table S5, *p* < 0.05). A positive correlation in occurrence of *Pseudomonadaceae* OTUs and presence of ARGs conferring resistance to multidrug, triclosan, polymyxin, quinolone, bacitracin, aminoglyocide, peptide, β-lactam, and fosfomycin were noted (Supplementary Table S5; *p* < 0.05). *Enterobacteriaceae* OTU occurrence was positively correlated with detection of peptide, multidrug, β-lactam, polymyxin, quinolone, triclosan, and aminoglycoside genes (Supplementary Table S5; *p* < 0.05). In addition, compost-associated bacterial families – *Bacillaceae, Thermoactinomycetaceae, Streptomycetaceae, Paenibacillaceae, Nocardiopsaceae*, and *Clostridiaceae* exhibited significant positive correlation with some ARGs detected on lettuce phyllosphere (Supplementary Table S5, *p* < 0.05). Interestingly, detection of *Carnobacteriaceae* on the lettuce phyllosphere increased with cold storage and was significantly correlated with ARGs conferring resistance to several antibiotics (Supplementary Table S5, *p* < 0.05).

### Irradiation and Washing Influences Dynamics of the ARG Resistome on Romaine Lettuce

**FIGURE 5:**
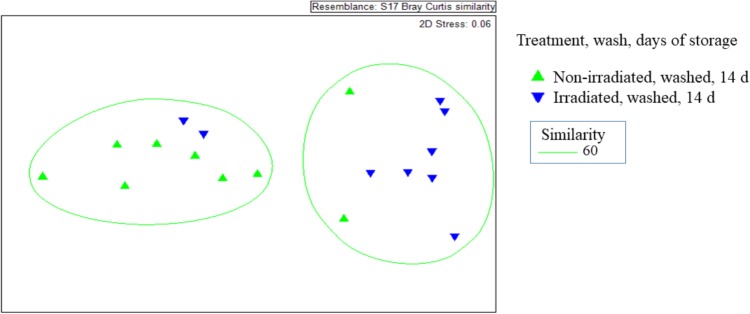
Non-metric multidimensional scales of Bray-Curtis distance matrices of ARGs found on irradiated and non-irradiated, sodium hypochlorite washed lettuce surfaces on day 14 of storage. Each point represents one lettuce sample (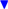 irradiated and washed, *n* = 9) and each (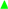 non-irradiated and washed; *n* = 9). ANOSIM, *R* = 0.406, *p* = 0.008 indicates distinct ARG profiles between non-irradiated and irradiated lettuce stored at 4°C for 14 days.

The β-diversity of resistome, as represented by the Bray-Curtis similarity of ARG occurrence, was significantly affected by irradiation (Figure 5). Non-irradiated lettuce carried distinct ARG profiles compared to irradiated lettuce (ANOSIM, *R* = 0.406, *p* = 0.008, Figure 5). Irradiation of lettuce resulted in significant decreases in relative abundance of six ARG classes compared to non-irradiated lettuce (Wilcoxon, *p* < 0.015, Table 3). The largest decreases in relative abundance of ARG classes (fold change in parentheses after class) on irradiated lettuce compared to non-irradiated lettuce were triclosan (4x), quinolones (3x), multidrug (2x), polymyxin (2x), and β-lactam (2x). ARG class conferring resistance to fosfomycin, which was detected at low relative abundance on non-irradiated lettuce, was not detected on irradiated lettuce (Table 3). Washing prior to irradiation was associated with further reductions to relative abundance of nine ARG classes encoding resistance (listed in order of largest change between unwashed and washed samples) to aminoglycoside, β-lactam, peptide, bacitracin, multidrug, sulfonamide, polymyxin, phenicol, and glycopeptide (*p* < 0.05, Wilcoxon; Table 3). In absence of irradiation, washing with sodium hypochlorite (50 ppm free chlorine) resulted in a 1.5 log reduction in total ARGs compared to unwashed lettuce. Significant decreases in relative abundance of only five ARG classes occurred, however, no significant decline was noted with the most abundant multidrug and triclosan classes, where irradiation was an effective strategy for reduction (Table 3, Wilcoxon, *p* < 0.05).

**Table 3:** Average relative abundance of ARGs on unprocessed and processed lettuce at Day 14.

Treatment ARG class	Non-irradiated unwashed day 14	Non-irradiated washed day 14	Irradiated unwashed day 14	Irradiated washed day 14
Multidrug	1.55	1.09	1.48***	0.47**
MLS	0.11	0.12	0.15	0.12
Triclosan	0.10	0.12	0.10	0.03**
Polymyxin	0.15	0.10	0.14***	0.05**
Glycopeptide	0.15	0.04*	0.02***	0.01
Aminoglycoside	0.13	0.05	0.15***	0.03
Tetracycline	0.08	0.04	0.08	0.06
Beta-lactam	0.08	0.04*	0.09***	0.02**
Trimethoprim	0.08	0.06	0.08	0.08
Quinolone	0.08	0.06	0.06	0.02**
Rifamycin	0.07	0.02	0.06	0.03
Peptide	0.05	0.04	0.04***	0.01
Phenicol	0.05	0.02*	0.05***	0.02
Bacitracin	0.06	0.02	0.04***	0.01
Fosfomycin	0.02	0.01	0.02	ND
Sulfonamide	0.03	ND^∗^	0.03***	0.01
Aminocoumarin	0.03	ND^∗^	0.02	ND


**^∗^ indicates statistical difference (p < 0.05, Wilcoxon) between unwashed and washed non-irradiated samples. ^∗∗^ indicates statistical difference (p < 0.05, Wilcoxon) between non-irradiated and irradiated washed samples. ^∗∗∗^ indicates statistical difference (p < 0.05, Wilcoxon) between unwashed and washed irradiated samples. MLS, Macrolide-lincosamide-streptogramin. ND indicates not detected.**

Further exploration of ARGs of elevated clinical concern revealed the presence of β-lactamases (*OXA*-50, *TEM*-17, *TEM*-91) and vancomycin-resistance (*van*A) in the lettuce resistome. *OXA*-50 and *TEM*-17 were detected on all lettuce samples dipped in ARB-inoculated compost, *TEM*-91 was detected in 70% of lettuce samples treated with ARB-inoculated compost, and *van*A was detected in 88% of all lettuce samples. Irradiation and washing did not significantly reduce the relative abundance of *OXA*-50, *TEM*-17, *TEM*-91, or *van*A (Supplementary Table S6).

## Discussion

Including a chlorine-based sanitizer in the wash water followed by MAP and cold temperature storage yielded small reductions in ARB and some classes of ARGs on lettuce. Substantial improvements in the reduction of human pathogens, levels of ARGs and their host bacterial communities were apparent after gamma irradiation treatment in combination with sanitizer washing, MAP, and cold storage. Gamma irradiation has been established as an effective control for *E. coli* O157:H7 on spinach, romaine and iceberg lettuce (Niemira, 2007, 2008). Even under circumstances where pathogens are part of leaf-surface biofilms, necessitating a higher treatment dose (D10 0.4–0.5 kGy) to achieve target reductions, irradiation remains an effective antimicrobial treatment (Niemira and Cooke, 2010). Results from this study characterize the broader changes to the non-culturable bacterial communities present on lettuce phyllosphere processed by gamma irradiation and stored for 14 days, a time period that is relevant for the produce industry. Previous studies have explored the effect of temperature, pH and chemical composition of food on the inactivation of culturable bacteria achieved by gamma irradiation in buffers, growth media, on meat products and other commodities (Monk et al., 1995; Sommers et al., 2002; Mañas and Pagán, 2005; Alvarez et al., 2006). In general, it has been reported that Gram negative (e.g., members of *Gammaproteobacteria*) and Gram positive (e.g., members of *Lactobacillales)* vegetative bacteria are more sensitive to irradiation than spore-forming bacteria (e.g., members of *Bacilli, Clostridia*) (Monk et al., 1995). Similar results were observed in the present study, where the greatest reductions in alpha-diversity corresponded to decreases in the *Proteobacteria* and increases in *Firmicutes*. The most significant changes were the reduction in *Pseudomonas*, *Escherichia*, and *Yersinia* in the *Gammaproteobacteria* and increase in *Clostridaceae*. The relative abundance of other bacterial taxa was more impacted by the time of storage, with significant decreases in *Actinobacteria* (10-fold), *Pseudomonadceae* (10-fold), and *Planococcaceae* (3-fold) and increases in the *Carnobacteriaceae* indicating the importance of growth of remaining the bacteria during the 14 days cold storage period to alter the community structure of the bagged lettuce. The range of radiation sensitivities and the propensity of different organisms to undergo repair, recovery and regrowth post-irradiation is well documented (Niemira et al., 2004). Irradiation was effective for reducing the inoculated strains of *E. coli* and *P. aeruginosa* during the 14 days. Members of the same families *Enterobacteriaceae* and *Pseudomonadceae* were also observed for non-inoculated lettuce indicating the ability of irradiation to reduce other species within these bacterial groups. These groups of bacteria, especially psychrotrphic pseudomonads, are responsible for vegetable tissue decay in ready to eat vegetables (Lee et al., 2013). The 16S rRNA amplicon sequencing also revealed that relative abundance of *Yersinia*, an under-recognized human pathogen, significantly decreased after irradiation. Species of *Yersinia*, chiefly *Y. pseudotuberculosis*, have been associated 47 illnesses, including one death in Finland attributed to modified atmosphere packaged iceberg lettuce (Nuorti et al., 2004).

A number of recent studies have detected antibiotic resistant bacteria on vegetable products at harvest or on retail product for purchase (Holzel et al., 2018). Bacteria from vegetables can carry ARGs commonly found in clinical isolates. For example, *Pseudomonas teesida* strains, isolated from packaged spinach, were shown to harbor genetic determinants associated with extended spectrum beta lactamase activity (ESBL Bla _CTX-M-15_) (Raphael et al., 2011). Plasmids, frequently associated with horizontal transfer of ARGs including IncP1 and IncQ, have been detected in gentamycin-resistant bacteria and oxytetracycline resistant bacteria isolated from lettuce (Rodriguez et al., 2006). Manure amendment to soil has been associated with increased levels of ARB and ARGs to soils and vegetables grown in those soils (Marti et al., 2013; Wind et al., 2018). In the present study, compost was used to serve as a source of ARGs and ARBs that may occur in the field. Genes encoding resistance to 22 classes of antibiotics were discovered. Extended-spectrum β-lactamase genes of potential clinical concern (*TEM-*17, *TEM*-91) and *OXA*-50 β-lactamase were detected on ARB-inoculated lettuce samples. *TEM* β-lactamase was present on the conjugative plasmid of inoculated antibiotic-resistant *E. coli* in this study that may result in the detection of *TEM* genes on inoculated lettuce samples. TEM-type ESBL genes have been detected on lettuce and ready-to-eat salads, and on lettuce grown on unmanured soils (Campos et al., 2013; Marti et al., 2013; Kim et al., 2015). TEM genes found in this study are resistant to antibiotic drug class penem, cephalosporin, monobactam, and penam (Jia et al., 2017). This study also detected *van*A, conferring resistance to vancomycin (a glycopeptide). This gene was not detected in compost used in this study (Williams, 2017). The *van*A gene cluster is commonly present on transposon, which can subsequently be associated with plasmid of Gram-positive cocci and has the potential of transfer to other Gram-positive bacteria (Cetinkaya et al., 2000; Freitas et al., 2016). The dissemination of *van*A in Gram positive bacteria, such as enterococci, staphylococci, and streptococci leads to an increase in nosocomial infections (Courvalin, 2006). These findings establish that additional strategies for reduction of clinical relevant ARGs of potential concern are required on lettuce. This establishes that vegetables, especially those consumed raw are an exposure route for ARB and ARGs that may influence the human gut microbiome (Sannes et al., 2008). Therefore, identifying strategies to reduce the occurrence of ARBs and ARGs on leafy vegetables may be a strategy to reduce overall risk to human health.

Implementation of practices designed to prevent the contamination of fresh fruits and vegetables with human pathogens is the first line of defense in regards to protecting the human gut microbiome. Strategies to reduce pathogens and ARBs by physical removal such as peeling can be effective for some types of produce but are not applicable for softer leafy greens. In this study, washing lettuce in water containing sodium hypochlorite achieved small reductions in the number of inoculated ARBs and reduced the relative abundance of five classes of ARGs. Further reductions were achieved by combining the washing with gamma- irradiation to decrease the relative abundance of ARGs encoding resistance to nine classes of antibiotics, including the multi-drug resistance class that was most abundant. Reductions in ARGs were not uniform across genes encoding resistance to all classes, likely due to carriage by Gram positive bacteria that are more resistant to gamma irradiation compared to Gram negative bacteria (Monk et al., 1995), including the inoculated *E. coli* O157:H7 and *Pseudomonas*. The reductions in ARG classes encoding resistance for multidrug, β-lactam, peptide, polymyxin, quinolone, and triclosan correlated with decreases in the *Enterobacteriaceae* and *Pseudomonadaceae.* Similar to this study, a positive correlation of the occurrence of *Pseudomonadaceae* with genes encoding resistance to multidrug and β-lactam antibiotics has also been reported for lettuce grown in manured soils (Marti et al., 2013; Zhu et al., 2017). This indicates that gamma irradiation may be a strategy to reduce commonly occurring ARGS associated with *Pseudomonadaceae.* In addition, several other classes of ARGs were correlated with occurrence of other bacterial groups (*Thermoactinomycetaceae, Nocardiopsaceae, Paenibacillaceae*, and *Bacillaceae)* that were significantly reduced by irradiation and refrigerated storage for 14 days. The presence of genes associated with resistance to MLS, pleuromutilin and tetracycline classes were positively associated with *Clostridiaceae* and *Carnobacteriaceae*, which were more prevalent in irradiated samples stored for 14 days. While the reduction in the relative abundance of ARGs was variable due to its presence in different host bacteria, irradiation was an effective strategy to reduce the total ARG copies/16S rRNA gene copies. To assure comparison amongst treatments, the equal amounts of DNA from each treatment were used for sequencing. In addition, to account for differences in sequencing depth the samples were rarified to 14,998,741 reads.

Metagenomics is a powerful tool for comparing overall trends associated with genes conferring resistance to different classes of antibiotics, however, the detection limit is fairly high and quantitative capability not as precise as qPCR. Metagenomic analysis revealed the presence of *tet*(A), which was known to be present in the inoculated strain of *P. aeruginosa*, only in one sample of unwashed, non-irradiated lettuce stored for 14 days and was not detected in other samples. However, *tet*(A) was detected in all samples using qPCR (Supplementary Table S3). While small decreases in absolute abundance of *tet*(A) were detected after washing, overall there was no statistically-significant difference in *tet*(A) copy number after irradiation. There was also no increase in number of *tet*(A) copies between days 1 and 14 samples that might indicate growth of the inoculated strain or acquisition of the gene by other bacteria that survived irradiation. *tet*(A) copies spotted as naked DNA from lysed cells containing a known concentration also did not change after irradiation, indicating that irradiation at its current dose (1ky) did not destroy or detectably damage the gene itself, but chiefly altered the ARG profiles by killing or preventing the replication of the host ARB.

## Conclusion

Bacterial communities comprising the lettuce phyllosphere are diverse and harbor a wide array of ARGs, potentially serving as a route of dissemination of antibiotic resistance from leafy vegetables to the human microbiota. With respect to antibiotic resistance and public health concern, the question remains in terms of whether this represents a substantial human health risk (Holzel et al., 2018). Irradiation and washing were found to act synergistically in reducing some ARB and ARGs on the lettuce phyllosphere, and therefore may be viable options for mitigating antibiotic resistance in the food chain. The metagenomic approach applied here provided broad profiling of how these factors influence a broad range of ARBs and ARGs, but deeper sequencing may be necessary to identify effects on more rare, but potentially clinically important, forms of antibiotic resistance. Appropriate models for translating resistome characterization to measures of human health risk are also needed. Judicial uses of antibiotics in livestock, combined with strategic manure management practices likely offer the greatest impact for reducing ARB and ARG loads on produce. The use of hurdles that may further reduce antibiotic resistant bacteria on produce may be warranted, especially for sensitive populations including the immunocompromised.

## Author Contributions

VD, GG, BN, RB, AP, LS, and MP contributed conception and design of the study. BN performed irradiation experiments. VD performed experiments and statistical analysis. GG performed some of metagenomic analyses. VD wrote the first draft of the manuscript. GG, VD, and MP wrote sections of the manuscript. All authors contributed to manuscript revision, read, and approved the submitted version.

## Conflict of Interest Statement

The authors declare that the research was conducted in the absence of any commercial or financial relationships that could be construed as a potential conflict of interest.

## Footnotes

1 http://bench.cs.vt.edu/MetaStorm/
