# Supplementary material for: Gamma Irradiation Influences the Survival and Regrowth of Antibiotic-Resistant Bacteria and Antibiotic-Resistance Genes on Romaine Lettuce
Source: Front Microbiol. 2019 Apr 9;10:710. doi: 10.3389/fmicb.2019.00710 (PMC6465624; doi:10.3389/fmicb.2019.00710)
Supplement: Supplementary file 1 [file Table_1.docx]

**SUPPLEMENTARY TABLES**

**Table S1**. Relative abundance (%) of bacterial taxa on compost-inoculated lettuce prior to washing and irradiation.

| ***Treatment***  ***Taxa*** | Unwashed Non-irradiated  Day 1 |
| --- | --- |
| **Phylum**  *Proteobacteria*  *Firmicutes*  *Actinobacteria*  *Other* | 42.90  36.30  13.25  7.55 |
| **Class**  *Bacilli*  *Gammaproteobacteria*  *Actinobacteria*  *Clostridia*  *Betaproteobacteria*  *Alphaproteobacteria*  *Other* | 30.45  28.15  13.20  10.95  3.15  3.10  8.25 |
| **Order**  *Bacillales*  *Pseudomonadales*  *Actinomycetales*  *Methylophilales*  *Enterobacteriales*  *Clostridiales*  *Xanthomonadales*  *Burkholderiales*  *Alteromonadales*  *Other* | 30.25  16.55  13.20  6.10  5.70  5.50  2.85  4.45  2.65  12.75 |
| **Family**  *Pseudomonadaceae*  *Planococcaceae*  *Nocardiopsaceae*  *Methylophilaceae*  *Enterobacteriaceae*  *Bacillaceae*  *Bacillales, other*  *Paenibacillaceae*  *Thermoactinomycetaceae*  *Clostridiaceae*  *Symbiobacteriaceae*  *Xanthomonadaceae*  *Other*  **Genus**  *Pseudomonas*  *Ureibacillus*  *Escherichia*  *Bacillus*  *Symbiobacterium*  *Thermoactinomyces*  *Clostridium*  *Yersinia*  *Enterobacteriaceae, other*  *Escherichia*  *Other* | 13.80  10.75  6.10  6.10  5.70  5.60  4.90  4.50  3.05  0.40  3.60  2.35  33.15  13.30  8.65  5.60  4.50  3.60  2.20  0.40  0.00  0.10  5.60  61.65 |

**Table S2**. Comparison of Chao1 and Shannon index of romaine lettuce within each treatment type. Connecting letters indicate significant differences among treatment types (p<0.05, Wilcoxon).

| Treatment Type | Lower limit | **Chao1**  Upper limit | Average + SE | Lower limit | **Shannon Index**  Upper limit | Average + SE |
| --- | --- | --- | --- | --- | --- | --- |
| **Compost** |  |  |  |  |  |  |
|  |  |  |  |  |  |  |
|  |  |  |  |  |  |  |
| **Wash Type** |  |  |  |  |  |  |
| Unwashed | 450.08 | 653.14 | 574.41 ± 22.73^A^ | 6.40 | 6.70 | 6.57 ± 0.03^A^ |
| Washed  (hypochlorite) | 107.20 | 621.43 | 323.42 ± 37.17^B^ | 0.61 | 6.36 | 3.54 ± 0.43^B^ |
|  |  |  |  |  |  |  |
| **Irradiation**  Non-irradiated  Irradiated | 114.11  107.20 | 640.23  653.14 | 371.05 ± 48.65^A^  410.22 ± 49.74^A^ | 1.41  0.61 | 6.64  6.69 | 4.48 ± 0.50^A^  4.28 ± 0.61^A^ |
| **Days of storage** |  |  |  |  |  |  |
| 1 | 418.37 | 653.14 | 545.99 ± 31.30^A^ | 4.98 | 6.70 | 6.23 ± 0.21^A^ |
| 14 | 107.20 | 612.02 | 334.25 ± 39.63^B^ | 0.61 | 6.60 | 3.67 ± 0.50^B^ |
|  |  |  |  |  |  |  |
|  |  |  |  |  |  |  |
|  |  |  |  |  |  |  |
|  |  |  |  |  |  |  |

**Table S3**. Table showing average relative abundance of *tet*(A) (*tet*(A) gene copies/16S rRNA gene copies) and absolute abundance of *tet*(A) (*tet*(A) gene copies) on irradiated and non-irradiated as well as irradiated washed and unwashed samples.

Relative abundance Absolute abundance

| Treatment | Lettuce dipped in dairy compost and ARB  Day1 | Lettuce dipped in dairy compost and ARB Day 14 |  | Lettuce dipped in dairy compost and ARB day 1 | Lettuce dipped in dairy compost and ARB day 14 |
| --- | --- | --- | --- | --- | --- |
| Non-irradiated washed  Irradiated  Washed  Irradiated unwashed | 1.45 x 10^-4^A  1.05 x 10^-3^A  7.13 x 10^-3^A | 1.86 x 10^-4^A  3.66 x 10^-4^A  3.68 x 10^-3^A |  | 1.56 x 10^4^B  1.70 x 10^4^B  3.18 x 10^5^B | 9.73 x 10^3^B  7.18 x 10^3^B  1.55 x 10^5^B |

-Similar letters indicate no statistical differences separately between relative abundance and absolute abundance between treatment type and across days of storage (Wilcoxon, p>0.05)

**Table S4**. Table showing average relative abundance of *tet*(A) (*tet*(A) gene copies/16S rRNA gene copies) and absolute abundance of *tet*(A) (*tet*(A) gene copies) on irradiated and non-irradiated lettuce samples spot inoculated with *P. aeruginosa* DNA.

Treatment Relative abundance Absolute abundance

| Spot inoculated | Day 1 | Day 14 |  | Day 1 | Day 14 |
| --- | --- | --- | --- | --- | --- |
| Non- irradiated  Irradiated | 1.89 x 10^-1^a  3.37 x 10^-2^a | 8.07 x 10^-1^a  2.11 x 10^-1^a |  | 1.47 x 10^5^b  2.72 x 10^5^b | 8.61 x 10^5^b  1.95 x 10^5^b |

-Similar letters indicate no statistical differences separately between relative abundance and absolute abundance between treatment type and across days of storage (Wilcoxon, p>0.05)

**Table S5**. Spearman’s rank correlation coefficient between relative abundance of ARG classes and bacterial families detected on lettuce phyllosphere.

| **Gene class** | **Family** | **Spearman p** | **Prob>p** |
| --- | --- | --- | --- |
| aminocoumarin | *Pseudomonadaceae* | -0.0265 | 0.9067 |
| aminoglycoside | *Pseudomonadaceae* | 0.6431 | 0.0012 |
| bacitracin | *Pseudomonadaceae* | 0.514 | 0.0144 |
| beta-lactam | *Pseudomonadaceae* | 0.7297 | 0.0001 |
| fosfomycin | *Pseudomonadaceae* | 0.7483 | <.0001 |
| glycopeptide | *Pseudomonadaceae* | -0.0028 | 0.9901 |
| MLS | *Pseudomonadaceae* | -0.1519 | 0.4998 |
| multidrug | *Pseudomonadaceae* | 0.8645 | <.0001 |
| peptide | *Pseudomonadaceae* | 0.6714 | 0.0006 |
| phenicol | *Pseudomonadaceae* | 0.2953 | 0.1821 |
| pleuromutilin | *Pseudomonadaceae* | -0.3208 | 0.1455 |
| polymyxin | *Pseudomonadaceae* | 0.8058 | <.0001 |
| quinolone | *Pseudomonadaceae* | 0.7764 | <.0001 |
| rifamycin | *Pseudomonadaceae* | -0.0028 | 0.9901 |
| sulfonamide | *Pseudomonadaceae* | 0.3559 | 0.1041 |
| tetracycline | *Pseudomonadaceae* | -0.0604 | 0.7894 |
| triclosan | *Pseudomonadaceae* | 0.8306 | <.0001 |
| trimethoprim | *Pseudomonadaceae* | -0.1259 | 0.5766 |
| aminocoumarin | *Enterobacteriaceae* | -0.0548 | 0.8087 |
| aminoglycoside | *Enterobacteriaceae* | 0.6318 | 0.0016 |
| bacitracin | *Enterobacteriaceae* | 0.6315 | 0.0016 |
| beta-lactam | *Enterobacteriaceae* | 0.6315 | 0.0016 |
| fosfomycin | *Enterobacteriaceae* | 0.6017 | 0.0031 |
| glycopeptide | *Enterobacteriaceae* | -0.0333 | 0.883 |
| MLS | *Enterobacteriaceae* | -0.1575 | 0.4838 |
| multidrug | *Enterobacteriaceae* | 0.7775 | <.0001 |
| peptide | *Enterobacteriaceae* | 0.8419 | <.0001 |
| phenicol | *Enterobacteriaceae* | 0.2027 | 0.3656 |
| pleuromutilin | *Enterobacteriaceae* | -0.0175 | 0.9384 |
| polymyxin | *Enterobacteriaceae* | 0.7595 | <.0001 |
| quinolone | *Enterobacteriaceae* | 0.677 | 0.0005 |
| rifamycin | *Enterobacteriaceae* | -0.0028 | 0.9901 |
| sulfonamide | *Enterobacteriaceae* | 0.2966 | 0.1802 |
| tetracycline | *Enterobacteriaceae* | -0.0525 | 0.8165 |
| triclosan | *Enterobacteriaceae* | 0.5347 | 0.0103 |
| trimethoprim | *Enterobacteriaceae* | 0.0627 | 0.7817 |
| aminocoumarin | *Nocardiopsaceae* | 0.6273 | 0.0018 |
| aminoglycoside | *Nocardiopsaceae* | 0.3721 | 0.0881 |
| bacitracin | *Nocardiopsaceae* | 0.6394 | 0.0014 |
| beta-lactam | *Nocardiopsaceae* | 0.2502 | 0.2614 |
| fosfomycin | *Nocardiopsaceae* | 0.1523 | 0.4987 |
| glycopeptide | *Nocardiopsaceae* | 0.5042 | 0.0167 |
| MLS | *Nocardiopsaceae* | 0.0085 | 0.9702 |
| multidrug | *Nocardiopsaceae* | 0.2468 | 0.2683 |
| peptide | *Nocardiopsaceae* | 0.4094 | 0.0585 |
| phenicol | *Nocardiopsaceae* | 0.5031 | 0.017 |
| pleuromutilin | *Nocardiopsaceae* | 0.2095 | 0.3493 |
| polymyxin | *Nocardiopsaceae* | 0.266 | 0.2316 |
| quinolone | *Nocardiopsaceae* | 0.2648 | 0.2336 |
| rifamycin | *Nocardiopsaceae* | 0.668 | 0.0007 |
| sulfonamide | *Nocardiopsaceae* | 0.5971 | 0.0033 |
| tetracycline | *Nocardiopsaceae* | 0.4873 | 0.0214 |
| triclosan | *Nocardiopsaceae* | -0.0954 | 0.6727 |
| trimethoprim | *Nocardiopsaceae* | 0.2592 | 0.2441 |
| aminocoumarin | *Carnobacteriaceae* | -0.6217 | 0.002 |
| aminoglycoside | *Carnobacteriaceae* | -0.729 | 0.0001 |
| bacitracin | *Carnobacteriaceae* | -0.5699 | 0.0056 |
| beta-lactam | *Carnobacteriaceae* | -0.6964 | 0.0003 |
| fosfomycin | *Carnobacteriaceae* | -0.5823 | 0.0045 |
| glycopeptide | *Carnobacteriaceae* | -0.7177 | 0.0002 |
| MLS | *Carnobacteriaceae* | 0.4421 | 0.0394 |
| multidrug | *Carnobacteriaceae* | -0.607 | 0.0027 |
| peptide | *Carnobacteriaceae* | -0.3642 | 0.0956 |
| phenicol | *Carnobacteriaceae* | -0.607 | 0.0027 |
| pleuromutilin | *Carnobacteriaceae* | 0.3592 | 0.1006 |
| polymyxin | *Carnobacteriaceae* | -0.441 | 0.0399 |
| quinolone | *Carnobacteriaceae* | -0.6714 | 0.0006 |
| rifamycin | *Carnobacteriaceae* | -0.6544 | 0.001 |
| sulfonamide | *Carnobacteriaceae* | -0.7311 | 0.0001 |
| tetracycline | *Carnobacteriaceae* | -0.0785 | 0.7285 |
| triclosan | *Carnobacteriaceae* | -0.5844 | 0.0043 |
| trimethoprim | *Carnobacteriaceae* | 0.3834 | 0.0782 |
| aminocoumarin | *Bacillaceae* | 0.5404 | 0.0094 |
| aminoglycoside | *Bacillaceae* | 0.1214 | 0.5905 |
| bacitracin | *Bacillaceae* | 0.3106 | 0.1594 |
| beta-lactam | *Bacillaceae* | 0.1271 | 0.573 |
| fosfomycin | *Bacillaceae* | -0.053 | 0.8147 |
| glycopeptide | *Bacillaceae* | 0.3789 | 0.0821 |
| MLS | *Bacillaceae* | 0.3269 | 0.1375 |
| multidrug | *Bacillaceae* | 0.0186 | 0.9344 |
| peptide | *Bacillaceae* | 0.1542 | 0.4934 |
| phenicol | *Bacillaceae* | 0.4647 | 0.0293 |
| pleuromutilin | *Bacillaceae* | 0.5993 | 0.0032 |
| polymyxin | *Bacillaceae* | 0.1316 | 0.5595 |
| quinolone | *Bacillaceae* | -0.0954 | 0.6727 |
| rifamycin | *Bacillaceae* | 0.467 | 0.0284 |
| sulfonamide | *Bacillaceae* | 0.3473 | 0.1132 |
| tetracycline | *Bacillaceae* | 0.6601 | 0.0008 |
| triclosan | *Bacillaceae* | -0.2434 | 0.2751 |
| trimethoprim | *Bacillaceae* | 0.5415 | 0.0092 |
| aminocoumarin | *Burkholderiaceae* | -0.4625 | 0.0302 |
| aminoglycoside | *Burkholderiaceae* | 0.0785 | 0.7285 |
| bacitracin | *Burkholderiaceae* | -0.1971 | 0.3793 |
| beta-lactam | *Burkholderiaceae* | 0.0734 | 0.7454 |
| fosfomycin | *Burkholderiaceae* | 0.3023 | 0.1716 |
| glycopeptide | *Burkholderiaceae* | -0.4218 | 0.0506 |
| macrolide-lincosamide-streptogramin | *Burkholderiaceae* | -0.1575 | 0.4838 |
| multidrug | *Burkholderiaceae* | 0.2784 | 0.2097 |
| peptide | *Burkholderiaceae* | -0.0412 | 0.8555 |
| phenicol | *Burkholderiaceae* | -0.1654 | 0.4619 |
| pleuromutilin | *Burkholderiaceae* | -0.3897 | 0.073 |
| polymyxin | *Burkholderiaceae* | 0.2321 | 0.2987 |
| quinolone | *Burkholderiaceae* | 0.2242 | 0.3159 |
| rifamycin | *Burkholderiaceae* | -0.5392 | 0.0096 |
| sulfonamide | *Burkholderiaceae* | -0.1169 | 0.6044 |
| tetracycline | *Burkholderiaceae* | -0.205 | 0.3602 |
| triclosan | *Burkholderiaceae* | 0.4263 | 0.0479 |
| trimethoprim | *Burkholderiaceae* | -0.1383 | 0.5392 |
| aminocoumarin | *Pseudonocardiaceae* | 0.6251 | 0.0019 |
| aminoglycoside | *Pseudonocardiaceae* | 0.319 | 0.1479 |
| bacitracin | *Pseudonocardiaceae* | 0.636 | 0.0015 |
| beta-lactam | *Pseudonocardiaceae* | 0.2271 | 0.3096 |
| fosfomycin | *Pseudonocardiaceae* | 0.0753 | 0.7392 |
| glycopeptide | *Pseudonocardiaceae* | 0.4929 | 0.0198 |
| macrolide-lincosamide-streptogramin | *Pseudonocardiaceae* | 0.004 | 0.9861 |
| multidrug | *Pseudonocardiaceae* | 0.205 | 0.3602 |
| peptide | *Pseudonocardiaceae* | 0.4218 | 0.0506 |
| phenicol | *Pseudonocardiaceae* | 0.4353 | 0.0429 |
| pleuromutilin | *Pseudonocardiaceae* | 0.2739 | 0.2173 |
| polymyxin | *Pseudonocardiaceae* | 0.2264 | 0.3109 |
| quinolone | *Pseudonocardiaceae* | 0.2242 | 0.3159 |
| rifamycin | *Pseudonocardiaceae* | 0.6635 | 0.0008 |
| sulfonamide | *Pseudonocardiaceae* | 0.5737 | 0.0052 |
| tetracycline | *Pseudonocardiaceae* | 0.485 | 0.0221 |
| triclosan | *Pseudonocardiaceae* | -0.1451 | 0.5194 |
| trimethoprim | *Pseudonocardiaceae* | 0.301 | 0.1735 |
| aminocoumarin | *Thermoactinomycetaceae* | 0.9097 | <.0001 |
| aminoglycoside | *Thermoactinomycetaceae* | 0.4975 | 0.0185 |
| bacitracin | *Thermoactinomycetaceae* | 0.4982 | 0.0183 |
| beta-lactam | *Thermoactinomycetaceae* | 0.5027 | 0.0171 |
| fosfomycin | *Thermoactinomycetaceae* | 0.276 | 0.2137 |
| glycopeptide | *Thermoactinomycetaceae* | 0.8442 | <.0001 |
| macrolide-lincosamide-streptogramin | *Thermoactinomycetaceae* | 0.2072 | 0.3548 |
| multidrug | *Thermoactinomycetaceae* | 0.3066 | 0.1652 |
| peptide | *Thermoactinomycetaceae* | 0.2005 | 0.3711 |
| phenicol | *Thermoactinomycetaceae* | 0.7595 | <.0001 |
| pleuromutilin | *Thermoactinomycetaceae* | 0.0593 | 0.7932 |
| polymyxin | *Thermoactinomycetaceae* | 0.284 | 0.2002 |
| quinolone | *Thermoactinomycetaceae* | 0.2693 | 0.2255 |
| rifamycin | *Thermoactinomycetaceae* | 0.764 | <.0001 |
| sulfonamide | *Thermoactinomycetaceae* | 0.7522 | <.0001 |
| tetracycline | *Thermoactinomycetaceae* | 0.7086 | 0.0002 |
| triclosan | *Thermoactinomycetaceae* | 0.1203 | 0.5939 |
| trimethoprim | *Thermoactinomycetaceae* | 0.0096 | 0.9662 |
| aminocoumarin | *Micrococcaceae* | 0.4963 | 0.0188 |
| aminoglycoside | *Micrococcaceae* | 0.4353 | 0.0429 |
| bacitracin | *Micrococcaceae* | 0.5626 | 0.0064 |
| beta-lactam | *Micrococcaceae* | 0.4507 | 0.0353 |
| fosfomycin | *Micrococcaceae* | 0.4688 | 0.0278 |
| glycopeptide | *Micrococcaceae* | 0.4478 | 0.0366 |
| macrolide-lincosamide-streptogramin | *Micrococcaceae* | -0.2829 | 0.2021 |
| multidrug | *Micrococcaceae* | 0.4896 | 0.0207 |
| peptide | *Micrococcaceae* | 0.301 | 0.1735 |
| phenicol | *Micrococcaceae* | 0.4568 | 0.0326 |
| pleuromutilin | *Micrococcaceae* | -0.4123 | 0.0565 |
| polymyxin | *Micrococcaceae* | 0.3665 | 0.0935 |
| quinolone | *Micrococcaceae* | 0.4986 | 0.0182 |
| rifamycin | *Micrococcaceae* | 0.4342 | 0.0435 |
| sulfonamide | *Micrococcaceae* | 0.5595 | 0.0068 |
| tetracycline | *Micrococcaceae* | 0.1835 | 0.4137 |
| triclosan | *Micrococcaceae* | 0.5302 | 0.0111 |
| trimethoprim | *Micrococcaceae* | -0.2377 | 0.2867 |
| aminocoumarin | *Flavobacteriaceae* | 0.3845 | 0.0772 |
| aminoglycoside | *Flavobacteriaceae* | 0.4026 | 0.0632 |
| bacitracin | *Flavobacteriaceae* | 0.5292 | 0.0113 |
| beta-lactam | *Flavobacteriaceae* | 0.4586 | 0.0318 |
| fosfomycin | *Flavobacteriaceae* | 0.5207 | 0.013 |
| glycopeptide | *Flavobacteriaceae* | 0.3382 | 0.1237 |
| MLS | *Flavobacteriaceae* | -0.1643 | 0.465 |
| multidrug | *Flavobacteriaceae* | 0.5178 | 0.0136 |
| peptide | *Flavobacteriaceae* | 0.52 | 0.0131 |
| phenicol | *Flavobacteriaceae* | 0.4455 | 0.0377 |
| pleuromutilin | *Flavobacteriaceae* | -0.1118 | 0.6203 |
| polymyxin | *Flavobacteriaceae* | 0.467 | 0.0284 |
| quinolone | *Flavobacteriaceae* | 0.4579 | 0.0321 |
| rifamycin | *Flavobacteriaceae* | 0.4184 | 0.0526 |
| sulfonamide | *Flavobacteriaceae* | 0.5851 | 0.0042 |
| tetracycline | *Flavobacteriaceae* | 0.1395 | 0.5359 |
| triclosan | *Flavobacteriaceae* | 0.4658 | 0.0289 |
| trimethoprim | *Flavobacteriaceae* | 0.0175 | 0.9384 |
| aminocoumarin | *Comamonadaceae* | 0.5291 | 0.0113 |
| aminoglycoside | *Comamonadaceae* | 0.4557 | 0.0331 |
| bacitracin | *Comamonadaceae* | 0.6744 | 0.0006 |
| beta-lactam | *Comamonadaceae* | 0.4117 | 0.0569 |
| fosfomycin | *Comamonadaceae* | 0.3992 | 0.0657 |
| glycopeptide | *Comamonadaceae* | 0.4478 | 0.0366 |
| MLS | *Comamonadaceae* | 0.0864 | 0.7023 |
| multidrug | *Comamonadaceae* | 0.4094 | 0.0585 |
| peptide | *Comamonadaceae* | 0.5167 | 0.0138 |
| phenicol | *Comamonadaceae* | 0.5359 | 0.0102 |
| pleuromutilin | *Comamonadaceae* | 0.0068 | 0.9761 |
| polymyxin | *Comamonadaceae* | 0.4207 | 0.0512 |
| quinolone | *Comamonadaceae* | 0.3427 | 0.1184 |
| rifamycin | *Comamonadaceae* | 0.5754 | 0.0051 |
| sulfonamide | *Comamonadaceae* | 0.6108 | 0.0025 |
| tetracycline | *Comamonadaceae* | 0.4015 | 0.064 |
| triclosan | *Comamonadaceae* | 0.1813 | 0.4195 |
| trimethoprim | *Comamonadaceae* | 0.1169 | 0.6045 |
| aminocoumarin | *Enterococcaceae* | -0.546 | 0.0086 |
| aminoglycoside | *Enterococcaceae* | -0.6217 | 0.002 |
| bacitracin | *Enterococcaceae* | -0.4756 | 0.0253 |
| beta-lactam | *Enterococcaceae* | -0.5812 | 0.0046 |
| fosfomycin | *Enterococcaceae* | -0.5121 | 0.0148 |
| glycopeptide | *Enterococcaceae* | -0.6578 | 0.0009 |
| MLS | *Enterococcaceae* | 0.476 | 0.0251 |
| multidrug | *Enterococcaceae* | -0.5065 | 0.0162 |
| peptide | *Enterococcaceae* | -0.2851 | 0.1983 |
| phenicol | *Enterococcaceae* | -0.4929 | 0.0198 |
| pleuromutilin | *Enterococcaceae* | 0.4417 | 0.0396 |
| polymyxin | *Enterococcaceae* | -0.3168 | 0.1509 |
| quinolone | *Enterococcaceae* | -0.6002 | 0.0031 |
| rifamycin | *Enterococcaceae* | -0.6115 | 0.0025 |
| sulfonamide | *Enterococcaceae* | -0.6336 | 0.0015 |
| tetracycline | *Enterococcaceae* | 0.0525 | 0.8165 |
| triclosan | *Enterococcaceae* | -0.5415 | 0.0092 |
| trimethoprim | *Enterococcaceae* | 0.4647 | 0.0293 |
| aminocoumarin | *Paenibacillaceae* | 0.7877 | <.0001 |
| aminoglycoside | *Paenibacillaceae* | 0.3258 | 0.139 |
| bacitracin | *Paenibacillaceae* | 0.4733 | 0.0261 |
| beta-lactam | *Paenibacillaceae* | 0.2886 | 0.1927 |
| fosfomycin | *Paenibacillaceae* | 0.1329 | 0.5555 |
| glycopeptide | *Paenibacillaceae* | 0.6928 | 0.0004 |
| macrolide-lincosamide-streptogramin | *Paenibacillaceae* | 0.223 | 0.3184 |
| multidrug | *Paenibacillaceae* | 0.1553 | 0.4902 |
| peptide | *Paenibacillaceae* | 0.2411 | 0.2797 |
| phenicol | *Paenibacillaceae* | 0.6273 | 0.0018 |
| pleuromutilin | *Paenibacillaceae* | 0.3474 | 0.1132 |
| polymyxin | *Paenibacillaceae* | 0.1779 | 0.4284 |
| quinolone | *Paenibacillaceae* | 0.1191 | 0.5974 |
| rifamycin | *Paenibacillaceae* | 0.7448 | <.0001 |
| sulfonamide | *Paenibacillaceae* | 0.657 | 0.0009 |
| tetracycline | *Paenibacillaceae* | 0.7098 | 0.0002 |
| triclosan | *Paenibacillaceae* | -0.0887 | 0.6948 |
| trimethoprim | *Paenibacillaceae* | 0.2716 | 0.2214 |
| aminocoumarin | *Planococcaceae* | 0.6906 | 0.0004 |
| aminoglycoside | *Planococcaceae* | 0.2614 | 0.2399 |
| bacitracin | *Planococcaceae* | 0.5287 | 0.0114 |
| beta-lactam | *Planococcaceae* | 0.1982 | 0.3765 |
| fosfomycin | *Planococcaceae* | 0.0148 | 0.9478 |
| glycopeptide | *Planococcaceae* | 0.5573 | 0.007 |
| MLS | *Planococcaceae* | 0.1779 | 0.4284 |
| multidrug | *Planococcaceae* | 0.1045 | 0.6436 |
| peptide | *Planococcaceae* | 0.2851 | 0.1983 |
| phenicol | *Planococcaceae* | 0.5054 | 0.0164 |
| pleuromutilin | *Planococcaceae* | 0.3112 | 0.1586 |
| polymyxin | *Planococcaceae* | 0.1462 | 0.5161 |
| quinolone | *Planococcaceae* | 0.0796 | 0.7247 |
| rifamycin | *Planococcaceae* | 0.6838 | 0.0005 |
| sulfonamide | *Planococcaceae* | 0.5418 | 0.0092 |
| tetracycline | *Planococcaceae* | 0.5573 | 0.007 |
| triclosan | *Planococcaceae* | -0.2208 | 0.3235 |
| trimethoprim | *Planococcaceae* | 0.2818 | 0.204 |
| aminocoumarin | *Streptomycetaceae* | 0.9119 | <.0001 |
| aminoglycoside | *Streptomycetaceae* | 0.4771 | 0.0247 |
| bacitracin | *Streptomycetaceae* | 0.5445 | 0.0088 |
| beta-lactam | *Streptomycetaceae* | 0.4778 | 0.0245 |
| fosfomycin | *Streptomycetaceae* | 0.2521 | 0.2577 |
| glycopeptide | *Streptomycetaceae* | 0.8498 | <.0001 |
| MLS | *Streptomycetaceae* | 0.2501 | 0.2615 |
| multidrug | *Streptomycetaceae* | 0.2761 | 0.2136 |
| peptide | *Streptomycetaceae* | 0.2456 | 0.2705 |
| phenicol | *Streptomycetaceae* | 0.7391 | <.0001 |
| pleuromutilin | *Streptomycetaceae* | 0.1265 | 0.5748 |
| polymyxin | *Streptomycetaceae* | 0.2626 | 0.2378 |
| quinolone | *Streptomycetaceae* | 0.2321 | 0.2987 |
| rifamycin | *Streptomycetaceae* | 0.7922 | <.0001 |
| sulfonamide | *Streptomycetaceae* | 0.6838 | 0.0004 |
| tetracycline | *Streptomycetaceae* | 0.7369 | <.0001 |
| triclosan | *Streptomycetaceae* | 0.0807 | 0.7209 |
| trimethoprim | *Streptomycetaceae* | 0.0615 | 0.7856 |
| aminocoumarin | *Clostridiaceae* | 0.214 | 0.3389 |
| aminoglycoside | *Clostridiaceae* | -0.1993 | 0.3738 |
| bacitracin | *Clostridiaceae* | -0.292 | 0.1873 |
| beta-lactam | *Clostridiaceae* | -0.1502 | 0.5045 |
| fosfomycin | *Clostridiaceae* | -0.3416 | 0.1197 |
| glycopeptide | *Clostridiaceae* | 0.1361 | 0.5459 |
| MLS | *Clostridiaceae* | 0.6206 | 0.0021 |
| multidrug | *Clostridiaceae* | -0.371 | 0.0892 |
| peptide | *Clostridiaceae* | -0.2851 | 0.1983 |
| phenicol | *Clostridiaceae* | 0.2705 | 0.2234 |
| pleuromutilin | *Clostridiaceae* | 0.6733 | 0.0006 |
| polymyxin | *Clostridiaceae* | -0.2038 | 0.3629 |
| quinolone | *Clostridiaceae* | -0.4704 | 0.0272 |
| rifamycin | *Clostridiaceae* | 0.1237 | 0.5835 |
| sulfonamide | *Clostridiaceae* | -0.0422 | 0.8521 |
| tetracycline | *Clostridiaceae* | 0.6149 | 0.0023 |
| triclosan | *Clostridiaceae* | -0.4433 | 0.0388 |
| trimethoprim | *Clostridiaceae* | 0.3642 | 0.0956 |

**Table S6**: Average relative abundance of potential clinical relevant ARGs on unprocessed and processed romaine lettuce.

| Treatment  ARGs | ARG class | Non-irradiated  Unwashed  Day 14 | Non-irradiated  Washed  Day 14 | Irradiated  Unwashed  Day 14 | Irradiated  washed  Day 14 | |
| --- | --- | --- | --- | --- | --- | --- |
| *TEM*-17 | β-lactam | 0.0086 | 0.0013 | 0.0190 | 0.0013 |  |
| *TEM*-91 | β-lactam | 0.0020 | 0.0001 | 0.0009 | 0.0004 |  |
| *OXA*-50 | β-lactam | 0.0280 | 0.0044 | 0.0287 | 0.0055 |  |
| *van*A | Glycopeptide | 0.0080 | 0.0018 | 0.0147 | 0.0056 |  |

-No significant statistical differences were reported in ARGs between unwashed and washed non-irradiated as well as and irradiated and non-irradiated washed lettuce (p>0.05, Wilcoxon).
